# Isolation and multilineage differentiation of bone marrow mesenchymal stem cells from abattoir-derived bovine fetuses

**DOI:** 10.1186/1746-6148-9-133

**Published:** 2013-07-05

**Authors:** Yennifer Cortes, Margarita Ojeda, Diego Araya, Fernando Dueñas, María S Fernández, Oscar A Peralta

**Affiliations:** 1 Departamento de Fomento de la Producción Animal, Facultad de Ciencias Veterinarias y Pecuarias, Universidad de Chile, Santiago, Chile; 2 Instituto de Ciencia Animal, Facultad de Medicina Veterinaria, Universidad Austral de Chile, Valdivia, Chile; 3 Departamento de Ciencias Biológicas Animales, Facultad de Ciencias Veterinarias y Pecuarias, Universidad de Chile, Santiago, Chile; 4 Department of Biomedical Sciences and Pathobiology, Virginia-Maryland Regional College of Veterinary Medicine, Virginia Polytechnic Institute and State University, Blacksburg, 24061-0442, Virginia, USA

**Keywords:** Mesenchymal stem cell, Bovine fetuses, Differentiation potential, Multipotency

## Abstract

**Background:**

Mesenchymal stem cells (MSC) are multipotent progenitor cells localized in the stromal compartment of the bone marrow (BM). The potential of MSC for mesenchymal differentiation has been well documented in different animal models predominantly on rodents. However, information regarding bovine MSC (bMSC) is limited, and the differentiation potential of bMSC derived from fetal BM remains unknown. In the present study we sought to isolate bMSC from abattoir-derived fetal BM and to characterize the multipotent and differentiation potential under osteogenic, chondrogenic and adipogenic conditions by quantitative and qualitative analyses.

**Results:**

Plastic-adherent bMSC isolated from fetal BM maintained a fibroblast-like morphology under monolayer culture conditions. These cells expressed high levels of MSC surface markers (*CD73*, *CD90*, and *CD105*) and low levels of hematopoietic surface markers (*CD34* and *CD45*). Culture of bMSC under osteogenic conditions during a 27-day period induced up-regulation of the osteocalcin (*OC*) gene expression and alkaline phosphatase (ALPL) activity, and promoted mineralization of the matrix. Increasing supplementation levels of ascorbic acid to culture media enhanced osteogenic differentiation of bMSC; whereas, reduction of FBS supplementation compromised osteogenesis. bMSC increased expression of cartilage-specific genes aggrecan (*ACAN*), collagen 2A1 (*COL2A1*) and SRY (sex-determining region Y) box 9 (*SOX9*) at Day 21 of chondrogenic differentiation. Treatment of bMSC with adipogenic factors increased levels of fatty acid-binding protein 2 (*AP2*) mRNA and accumulation of lipid vacuoles after 18 days of culture. *NANOG* mRNA levels in differentiating bMSC were not affected during adipogenic culture; however, osteogenic and chondrogenic conditions induced higher and lower levels, respectively.

**Conclusions:**

Our analyses revealed the potential multilineage differentiation of bMSC isolated from abattoir-derived fetal BM. *NANOG* mRNA pattern in differentiating bMSC varied according to differentiation culture conditions. The osteogenic differentiation of bMSC was affected by ascorbic acid and FBS concentrations in culture media. The simplicity of isolation and the differentiation potential suggest that bMSC from abattoir-derived fetal BM are appropriate candidate for investigating MSC biology and for eventual applications for regenerative therapy.

## Background

Mesenchymal stem cells (MSC) are multipotent progenitor cells that localize in the stromal compartment of the bone marrow (BM), where they support hematopoiesis and differentiate into mesenchymal lineages [1]. The potential of MSC to form bone, cartilage, and adipose tissues has been well documented both in vivo [2] and in vitro [3]. However, the plasticity of MSC is not limited to mesenchymal derivatives, since MSC have been induced to differentiate into mesodermal, neuroectodermal and endodermal lineages [1]. MSC can be directly isolated from bone marrow aspirates based on their ability to adhere to plastic when plated in monolayer culture, by flow cytometry and sorting separation or with micromagnetic beads by using specific antibodies for stem cells [4]. Thereafter, human multipotent stem cells derived from marrow stroma proliferate ex vivo to form a phenotypically homogeneous population of cells that express surface antigens markers CD73 (ecto-5′-nucleotidase), CD90 (Thy-1), CD105 (endoglin) and lack expression of CD45 (protein tyrosine phosphatase, receptor type, C), CD34 (CD34 molecule) and CD14 (CD14 molecule) [5].

The regulatory mechanisms which are essential for maintenance of MSC characteristics or triggering differentiation are only partially understood [6]. After in vitro differentiation of human MSC into the osteogenic lineage, evaluation of the osteocyte phenotype has identified cells positive for the bone extracellular matrix protein osteocalcin (OC) and the mineralization-associated enzyme alkaline phosphatase (ALPL) [3,7]. Similarly, induction of chondrogenic differentiation results in the majority of cells expressing cartilage-specific proteins including matrix markers aggrecan (ACAN) and collagen 2A1 (COL2A1), and essential transcription factor SRY (sex-determining region Y) box 9 (SOX9) [8]. Whereas, the adipogenic induction has been characterized in human and mouse MSC by increase in the expression of fatty acid peroxisome proliferation-activated receptor γ (PPARγ) and fatty acid-binding protein 2 (AP2) [3,9].

Several studies have evaluated the expression of differentiation markers in MSC linages; however, fewer reports have analyzed the expression of pluripotent genes during differentiation of MSC. The transcriptional regulator octamer-binding protein 4 (OCT4) along with co-regulators NANOG and SRY (sex-determining region Y) 2 (SOX2) coordinate a program of gene activity that suppresses differentiation and allows self-renewal in embryonic stem (ES) cells [10,11]. In MSC; however, levels of OCT4 are low [12] or inexistent [13] and its activity has been reported to be dispensable for MSC self-renewal [12]. While the role of NANOG in MSC has been investigated at lesser extent than OCT4, expression of NANOG has been detected in MSC, where ectopic overexpression increases the potential for osteogenic differentiation [14].

Cells with features of mesenchymal precursors have been isolated from the BM of many mammals, including laboratory rodents [2], humans [3], cats [15], dogs [16] and pigs [17]. Despite the wide relevance of the bovine experimental model in both in vivo and in vitro experiments, limited information regarding bovine MSC (bMSC) is available. Considering their similarities in organ size and physiology with humans and their longer life span in comparison with the traditional laboratory animal models, large animals are considered to be an excellent model for long-term experiments in regenerative medicine [18,19]. Thus, derivation of bESC would be invaluable for testing the efficiency and safety of these cells for future cell therapies and for the creation of human disease models. Cattle can give numerous advantages for making progress in clinical applications of MSC to human medicine, especially in musculoskeletal health problems [20-22]. Previous studies reported isolation and mesenchymal differentiation of bMSC from calf BM [22,23] and bovine umbilical cord [24,25]. However, differentiation potential of bMSC derived from other sources including fetal BM remains unknown. Human MSC isolated from fetal BM have been shown to have higher proliferative capacity, trilineage differentiation potential and lower immunogenicity compared to MSC from umbilical cord, adult BM or adipose tissue [26]. In particular, human fetal BM MSC had higher proliferative and osteogenic capacity than MSC derived from other ontological and anatomical origins, suggesting they are superior candidates for bone tissue engineering [27,28].

The development of large animal experimental models including cattle may open alternative strategies for investigating MSC physiology and eventual applications for regenerative therapy in human and veterinary medicine. Currently, animal BM is the most common source of MSC for clinical and research uses. In the present study, we used abattoir-derived bovine fetuses as an available and plentiful source of BM with the aim to obtain an abundant supply of bMSC and to minimize risk for donor and recipient. Our main objective was to isolate bMSC from abattoir-derived fetal BM and to characterize the multipotent and differentiation potential during in vitro osteogenic, chondrogenic and adipogenic differentiation by quantitative and qualitative analyses.

## Results

### Isolation of bone marrow MSC from abattoir-derived bovine fetuses

Isolation of bMSC from fetal BM was performed based on the capacity for plastic attachment under standard culture conditions that included DMEM media supplemented with 10% FBS. Colonies of fibroblast-like cells attached to the plastic were visualized at Days 5–6 after seeding. Cells exhibited characteristic spindle shape and polygonal morphology. Isolated cells were cultured for several weeks in monolayer and used for differentiation experiments after 4 to 5 passages.

### Mesenchymal cell surface markers and population doublings

**Figure 1:**
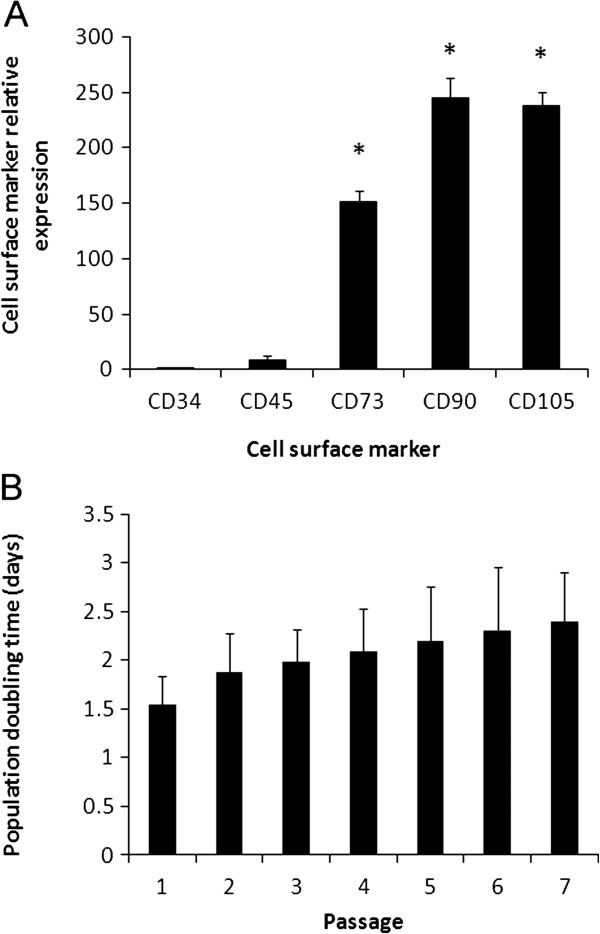
**Cell surface markers and population doublings of bMSC cultures isolated from fetal bone marrow. (A)** Higher (P < 0.001) levels of mRNA of MSC surface markers (*CD73*, *CD90*, and *CD105*) were detected in comparison to hematopoietic surface markers (*CD34* and *CD45*) levels. **(B)** PD time (days) were no significantly different among 7 consecutive culture passages. Superscripts (*) represent significant (P < 0.001) differences between MSC and hematopoietic surface markers.

Three independent cultures of bMSC were analyzed for the expression of mesenchymal and hematopoietic surface markers using quantitative-PCR (Q-PCR). bMSC expressed higher (P < 0.05) levels of MSC cell surface markers CD73, CD90, CD 105 (151.2-, 245.1-, 238.1-fold relative to CD34) compared to hematopoietic markers CD34 and CD45 (1- and 9-fold relative to CD34; Figure 1A). No significant differences were detected for population doublings (PD) between consecutive passages until passage 7 (Figure 1B).

### Osteogenic differentiation

**Figure 2:**
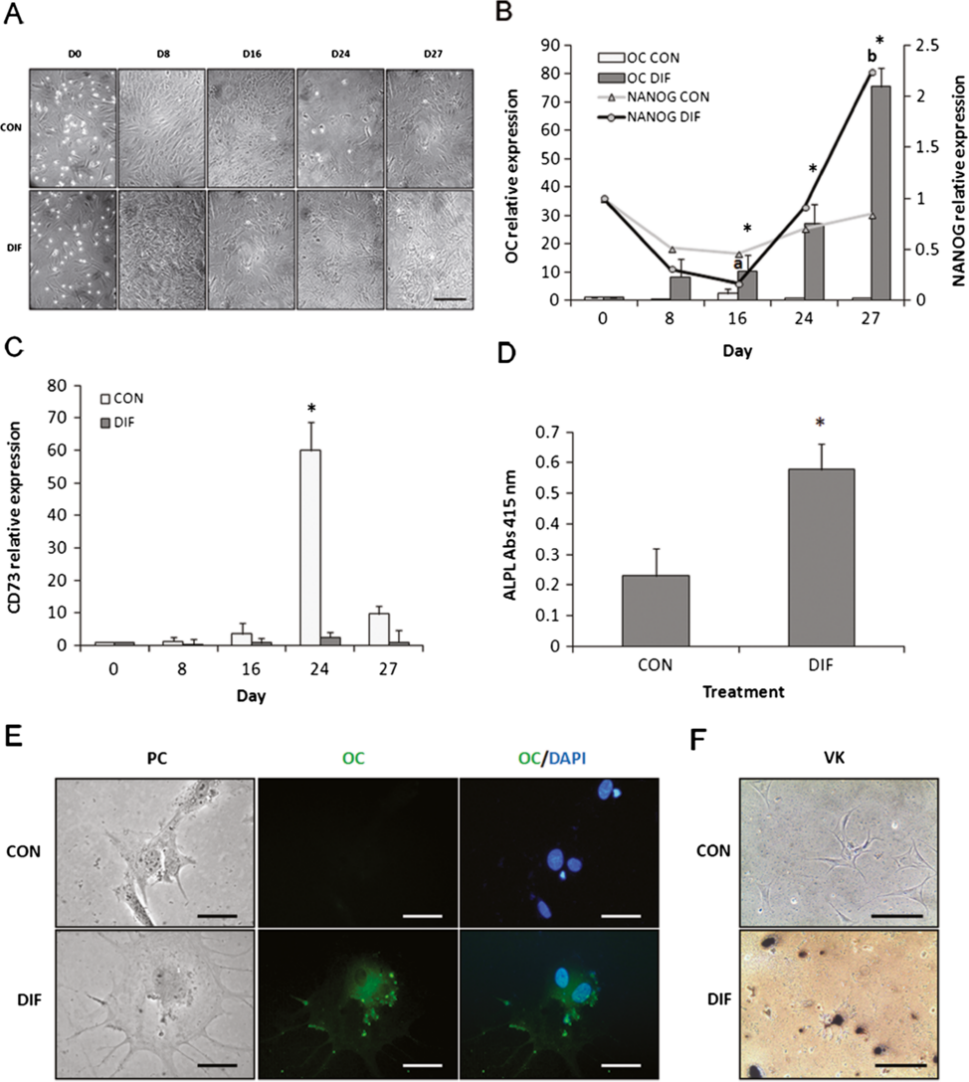
**Osteogenic differentiation of bMSC isolated from fetal bone marrow. (A)** Complex cell-interactions and extracellular matrix formation were observed in differentiating bMSC during a 27-Day culture period. **(B)** Higher (P < 0.05) levels of *OC* mRNA and ALPL activity were detected in differentiated bMSC at Days 16, 24 and 27 compared to untreated controls. *NANOG* mRNA levels increased (P < 0.05) from days 16 to 27 in differentiated mMSC. **(C)***CD73* mRNA expression was upregulated in untreated bMSC at Day 24 of osteogenic culture. **(D)** Activity of ALPL were higher (P < 0.05) in differentiated bMSC compared to control. **(E)** Osteogenesis was confirmed at Day 27 of culture by immunodetection of OC and **(F)** von Kossa staining of the matrix. Phase contrast (PC), OC and merged with DAPI for control and differentiation bMSC. Data are shown as mean ± standard errors. Superscripts (**a**,**b**,*) represent significant (P < 0.05) differences between treatment and sampling days. Bar scale: **(A)** 500 μm; **(E)** 20 μm; **(F)** 500 μm.

Supplementation of osteogenic factors during a 27-day period induced cell morphology changes that included formation of cell projections and development of intricate cell interactions and extracellular matrix (Figure 2A). *OC* mRNA levels significantly increased in differentiated bMSC at Day 16 of culture (10.2-fold relative to Day 0 vs. 2.4-fold in the untreated controls) and continue to rise through Day 27 (75.4-fold relative to Day 0 vs. 0.84-fold in the untreated controls, Figure 2B). At Day 27, *NANOG* mRNA levels significantly increased in differentiated bMSC compared to Day-16 control (2.2-fold vs. 0.2-fold relative to Day 0). *CD73* expression was up-regulated (P < 0.05) in untreated control bMSC at Day 24 of culture (Figure 2C). *ALPL* activity increased (P < 0.05) in differentiated bMSC at Day 27 of culture compared to untreated controls (0.58 vs. 0.23 Abs 415 nm, Figure 2D). OC expression was detected by immunofluorescence in differentiated bMSC at Day 27 of culture (Figure 2E). At this stage, an intense matrix mineralization was detected using von Kossa staining in differentiated bMSC cultures (Figure 2F).

### Effect of ascorbic acid and FBS supplementation on bMSC osteogenic differentiation

**Figure 3:**
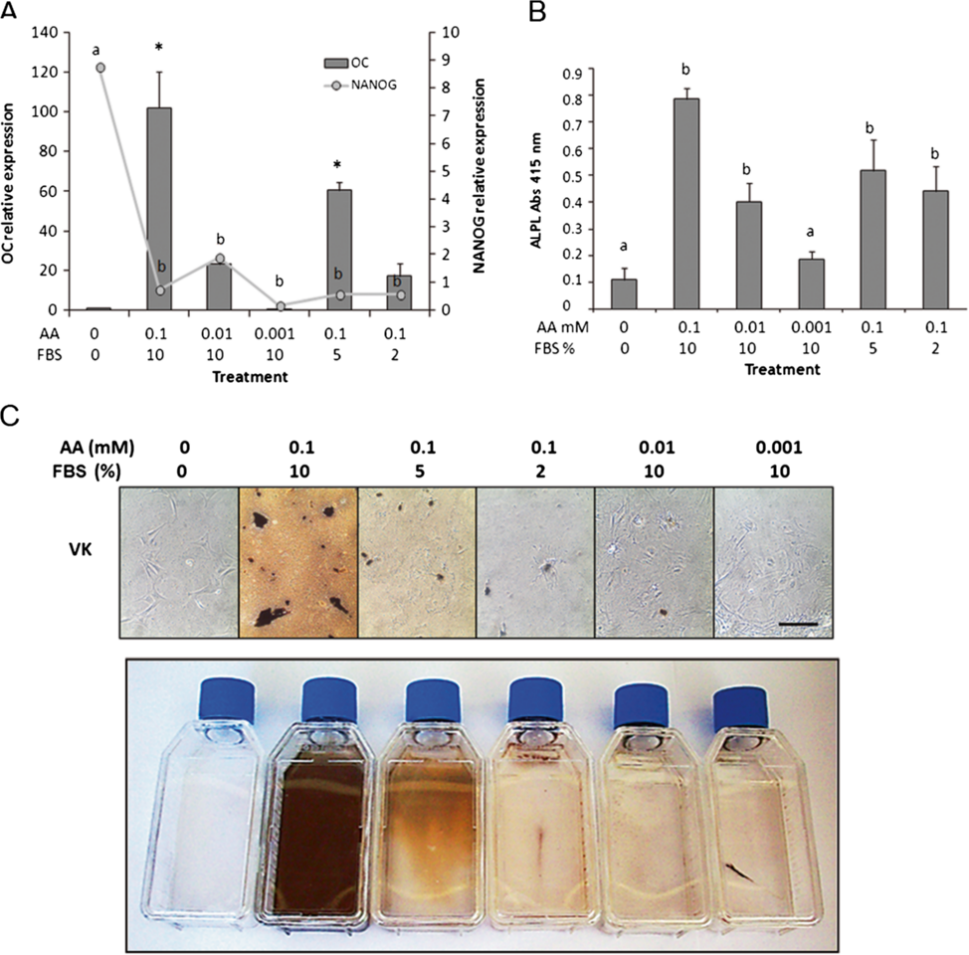
**Dose-dependent effect of the ascorbic acid and FBS supplementation during osteogenic differentiation of bMSC. (A)** Culture of bMSC under high concentrations of ascorbic acid (0.1 mM) in combination with 10% or 5% FBS resulted in higher (P < 0.05) *OC* mRNA and lower *NANOG* mRNA expression compared to other treatments. **(B)** ALPL activity decreased (P < 0.05) when acid ascorbic was eliminated or reduced to 0.001 mM. **(C)** von Kossa (VK) staining evidenced higher matrix mineralization in bMSC cultures supplemented with 0.1 mM ascorbic acid in combination with 10 and 5% FBS. Data are shown as mean ± standard errors. Superscripts (**a**,**b**,*) represent significant (P < 0.05) differences between treatment and sampling days. Bar scale: 500 μm.

Supplementation of 0.1 mM of ascorbic acid to bMSC cultures induced higher (P < 0.05) *OC* mRNA levels (102.1-fold relative to Day 0) compared to 0.01 mM (22.9-fold) and 0.001 mM (0.1-fold) concentrations at Day 27 of culture (Figure 3A). Reduction in FBS supplementation to 2% resulted in lower (P < 0.05) *OC* mRNA (17-fold relative to Day 0) compared to 10% (102.1-fold) and 5% (60.5-fold). *NANOG* mRNA levels decreased (P < 0.05) in bMSC supplemented with 0.1 mM (0.7-fold), 0.01 mM (1.9-fold) and 0.001 mM (0.2-fold) of ascorbic acid compared to untreated control cells (8.7-fold). Addition of 0.1 or 0.01 mM of ascorbic acid induced higher (P < 0.05) ALPL activity in bMSC compared to addition of 0.001 mM or no addition of ascorbic acid (Figure 3B). Treatment of 0.1 mM of ascorbic acid in combination with 10% or 5% FBS resulted in stronger von Kossa staining compared to other treatments (Figure 3C).

### Chondrogenic differentiation

**Figure 4:**
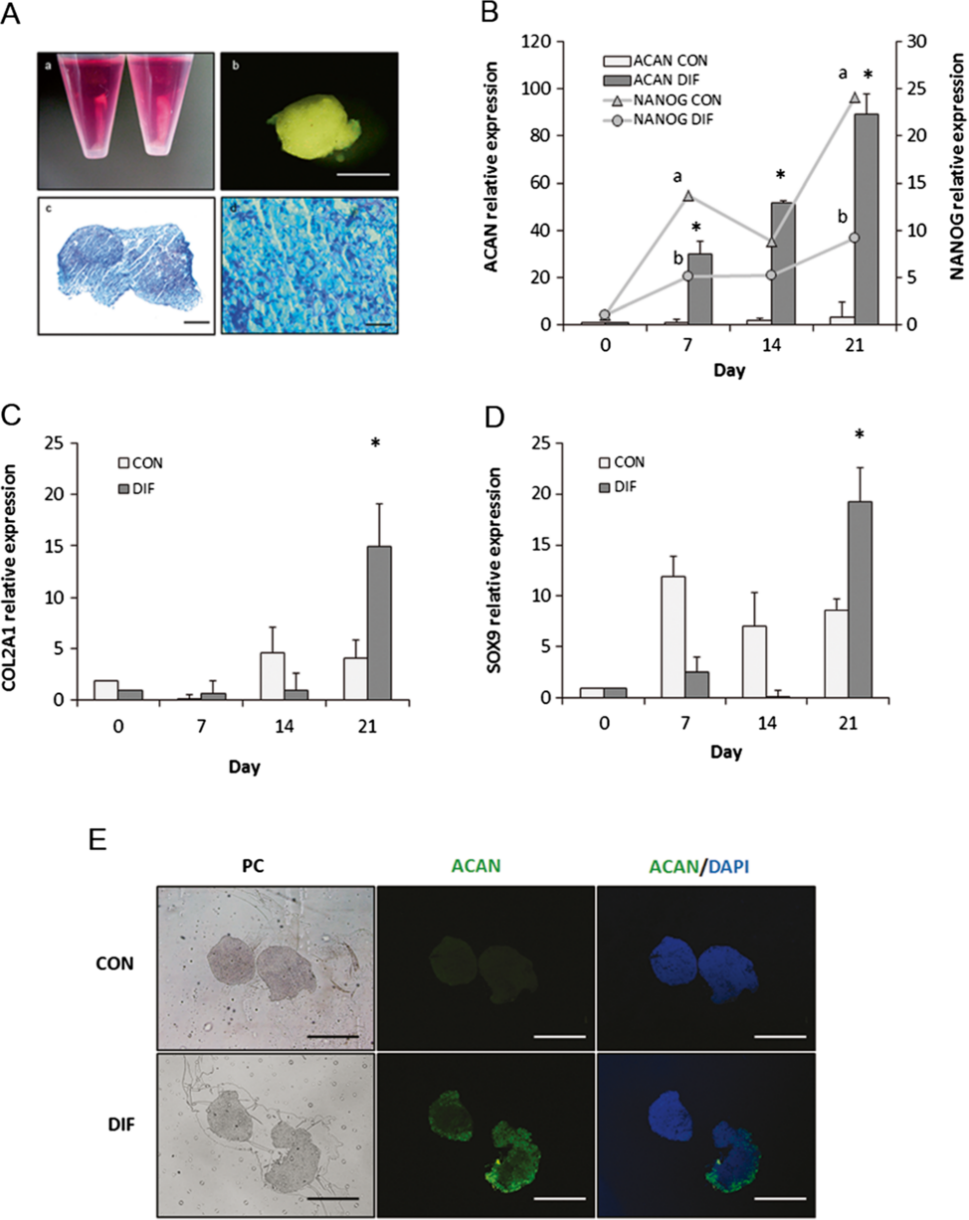
**Chondrogenic differentiation of bMSC isolated from fetal bone marrow. (A)** Culture of bMSC under differentiation media during 21 days resulted in cartilage pellet formation. **(a)** bMSC were differentiated using micromass culture system **(b)** formed cartilage pellet after 21 days of culture. **(c** and **d)** Presence of glycosaminoglycans was confirmed in histological sections of the pellet by alcian blue staining. **(B)** Levels of *ACAN* mRNA increased (P < 0.05) in differentiating bMSC at Days 7, 14 and 21compared to untreated controls. In contrast, lower (P < 0.05) levels of *NANOG* mRNA were detected in differentiating bMSC at Days 7 and 21compared to untreated controls. **(C) ***COL2A1* and **(D) ***SOX9* mRNA levels increased in differentiated bMSC at day 21 of culture. **(E)** ACAN was immunodetected in cartilage pellet at Day 21 of culture. Phase contrast (PC), ACAN, and merged with DAPI for control (treated with non-immune serum instead of primary antibody) and differentiation bMSC. Data are shown as mean ± standard errors. Superscripts (a,b,*) represent significant (P < 0.05) differences between treatment and sampling days. Bar scale: **(b)** 2 mm; **(c)** 200 μm; **(d)** 100 μm; **(C)**, 500 μm.

Cultured of bMSC under chondrogenic conditions using a micromass culture system for 21 days induced cartilage formation (Figure 4A). Glycosaminoglycan was detected using alcian blue staining in cartilage pellets at Day 21 of culture (Figure 4A). *ACAN* mRNA levels increased significantly in differentiated bMSC at Day 7 (29.8-fold relative to Day 0 vs. 0.9-fold in untreated control) and continued to rise through Day 21 of culture (89.2-fold relative to Day 0 vs. 3.5-fold in untreated control, Figure 4B). *NANOG* mRNA levels were lower (P < 0.05) in differentiating bMSC at Day 7 (5.1-fold relative to Day 0 vs. 13.7-fold in untreated control) and at Day 21 of culture (9.2-fold relative to Day 0 vs. 24.2-fold for untreated control, Figure 4B). *COL2A1* mRNA levels increased (P < 0.05) in differentiated bMSC at Day 21 of culture (14.9-fold relative to Day 0 vs. 4.1-fold in untreated control, Figure 4C). Similarly, *SOX9* mRNA levels significantly increased in differentiated bMSC at Day 21 of culture (19.2-fold relative to Day 0 vs. 8.5-fold in untreated control, Figure 4D). Immunoreactivity for ACAN was detected in histological sections of cartilage pellets at Day 21 of culture (Figure 4E).

### Adipogenic differentiation

**Figure 5:**
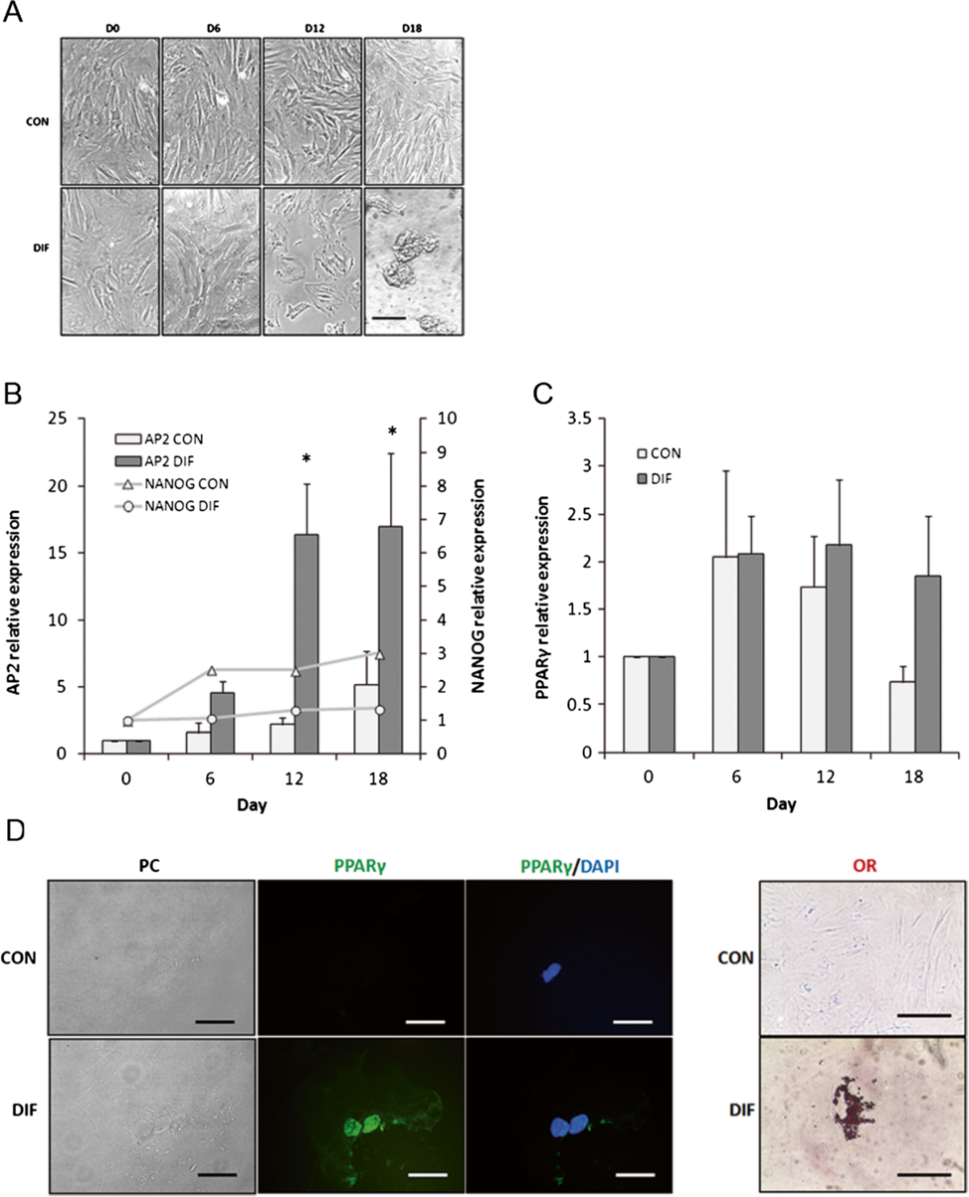
**Adipogenic differentiation of bMSC isolated from fetal bone marrow. (A)** Culture of bMSC under adipogenic conditions induced drastic morphology changes including formation of cell aggregates and accumulation of lipid vacuoles in the cytoplasm. **(B)***AP2* mRNA levels increased (P < 0.05) in differentiating bMSC at Days 12 and 18 of culture. **(C)** In contrast, expression levels of *PPAR*γ mRNA were similar between treatments. **(D)** PPARγ was immunodetected in differentiated bMSC but not in controls at Day 18 of culture. **(E)** Adipogenesis was confirmed by oil red (OR) staining of lipids vacuoles in differentiated bMSC at Day 18 of culture. Phase contrast, PPARγ and merged with DAPI for control and differentiation bMSC. Superscripts (a,b,*) represent significant (P < 0.05) differences between treatment and sampling days. Bar scale: **(A)** 500 μm; **(D)** 20 μm; **(E)** 500 μm.

Culture of bMSC under adipogenic conditions induced formation of cell aggregates and presence of lipid vacuoles in the cell cytoplasm (Figure 5A). *AP2* mRNA levels increased (P < 0.05) in differentiated bMSC at Day 12 (16.4-fold relative to Day 0 vs. 2.2-fold in untreated control) and at Day 18 (17- fold relative to Day 0 vs. 5.2-fold in untreated control) of culture (Figure 5B). No significant differences were detected in *NANOG* or *PPAR*γ mRNA levels between treatments or sampling days (Figures 5B,C). PPARγ was immunodetected associated to the nuclear cell compartment in differentiated bMSC at day 18 of culture (Figure 5D). Moreover, lipid vacuoles were stained with oil red in differentiated bMSC at Day 18 of culture (Figure 5E).

## Discussion

In the present study, bMSC were isolated from BM collected from abattoir-derived fetuses based on the capacity to adhere to plastic flasks under monolayer culture conditions. This method has been reported for isolation of MSC in various animal models including sheep [29], pig [17] and cattle [22]. Cell cultures isolated by this method are composed by a heterogeneous cell population that includes a mixture of progenitor cells with different degrees of commitment [30]. In our study, isolated bMSC maintained different cell morphologies from fibroblast-like, spindle-shaped cells to large flat cells. Despite this heterogeneous conformation, the bMSC cultures expressed high levels of mRNA of MSC specific surface markers *CD73*, *CD90* and *CD105* and scarce levels of hematopoietic cell surface markers *CD33* and *CD45*. A constant growth rate was detected in bMSC cultures for 7 consecutive passages. Similarly, bMSC collected from calves were reported to have constant growth rates until passage 10, followed by progressive reduction and culture senescence [23]. Reports in human adults MSC indicated that senescence begins at passage 3 [24], which suggests differences in proliferative potential according to developmental stage. Although the pluripotent potential of bMSC after more than 4 to 5 passages was not analyzed in the present study, it has been reported that bMSC collected from calves BM are able to maintain osteogenic differentiation potential for up to 15 passages [23].

The multipotent state and potential for multilineage differentiation of bMSC was evaluated using quantitative and qualitative analyses. Protocols used for differentiation of MSC have been previously reported in the same and other species [22,31]. Culture of bMSC during a 27-day period under osteogenic conditions induced complex cell morphology and extracellular matrix formation. *OC* mRNA levels and ALPL activity increased in differentiating bMSC compared to untreated controls. Osteogenesis was confirmed at Day 27 of differentiation by immunolabeling of OC and von Kossa staining of matrix mineralization. These results indicate that constant exposure to osteogenic medium induced a time-dependent osteogenesis in fetal bMSC. Previous studies reported similar osteogenic potential in bMSC isolated from calf BM and umbilical cord blood [22,25]. MSC derived from adult BM were reported to have equivalent osteogenic capacity in other species including horse [32], sheep [33] and pig [18]. Our data suggest that fetal bMSC bear comparable osteogenic potential to perinatal and postnatal MSC; however, a more comprehensive study is required in order to determine potential differences between ontological stages. CD73 is a membrane-bound glycoprotein involved in both signal transduction and ecto-5′-nucleotidase activity. Although CD73 is a key molecule for defining MSC [5], its specific role in MSC remains unknown. Recent studies have suggested that CD73 may be involved in MSC osteogenic differentiation via adenosine receptor (A2AAR and A2BAR) signaling [34]. In our study, *CD73* mRNA was detected in all stages of bMSC differentiation; however, at day 24 of culture differentiated bMSC expressed lower level of *CD73* mRNA compared to untreated controls. Similar reduction in CD73 expression has been reported after osteogenic differentiation in human MSC [35] suggesting that this protein may be regulated during osteogenic differentiation.

A dose-dependent experiment was performed to evaluate the effect of ascorbic acid and FBS supplementation in the osteogenic differentiation on bMSC. Ascorbic acid plays a key role as a cofactor in the post-translational modification of collagen molecules [36]. Various mesenchyme-derived cell types including osteoblasts, adipocytes, chondrocytes and odontoblasts increase collagen production and osteogenic potential after exposure to ascorbic acid [37]. In our study, culture of bMSC with high concentration of ascorbic acid (0.1 mM) resulted in higher *OC* mRNA levels and ALPL activity, and stronger mineralization of the matrix compared to lower concentration (0.001 mM). Moreover, *NANOG* mRNA levels were intensely down-regulated in bMSC treated with the same concentration. Supplementation of FBS to culture media has been shown to increase proliferation of MSC [38]. The effect of FBS alters the metabolic state of MSC and may also affect the terminal differentiation of MSC. In our study, reduction in FBS to 5% and 2% in bMSC culture media resulted in lower *OC* mRNA levels and less mineralization of the matrix compared to 10%. However, ALPL activity and *NANOG* mRNA levels were not affected by these treatments. Overall, these results indicate that osteogenic differentiation of bMSC may be enhanced by increasing ascorbic acid supplementation to media culture; whereas; reduction in FBS supplementation to 5% or 2% may compromise the osteogenic differentiation process.

Culture of bMSC for 21 days under chondrogenic conditions that included supplementation of TGFβ resulted in formation of a cartilage pellet. Up-regulation of cartilage-specific genes *ACAN*, *COL2A1* and *SOX9* evidenced the chondrogenic differentiation of bMSC. Alcian blue staining of glycosaminoglycans in histological sections of the cartilage pellet confirmed extracellular matrix formation. Comparable results were previously reported in other study using similar number of bMSC and concentration of TGFβ [39]. However, these authors also described spontaneous differentiation of bMSC cultured in TGFβ-free media. In our study, bMSC cultured in control media (high glucose DMEM) expressed low levels of *ACAN*, *COL2A1* and *SOX9* mRNA and were unable to undergo cartilage formation. Despite the effect of TGFβ was not evaluated in our study, our data indicate that media containing chondrogenic factors is required to induce differentiation of bMSC.

Adipogenic differentiation of bMSC was first characterized by observation of drastic morphology changes including formation of cell aggregates and accumulation of lipid vacuoles. Levels of *PPAR*γ mRNA in bMSC during adipogenesis were no significantly different between treatments. At Day 18 of adipogenic culture, mRNA levels of *PPAR*γ were 2.5-fold higher in differentiated compared to control bMSC. These results differ from a previous study using bMSC isolated from 2-to-6 months old calves, where *PPAR*γ mRNA levels were 8.4-fold higher in differentiated compared to control cells [22]. Differences in results between both studies may be associated to the limited developmental activation of the *PPAR*γ gene in fetal compared to calf bMSC, since preadipocyte differentiation and number of PPARγ positive cells have a dependent association with fetal age [40]. Despite reduced levels of *PPAR*γ mRNA, the PPARγ protein was immunodetected in the nuclei of differentiated bMSC at Day 18 of culture. Nuclear localization follows PPARγ activation in various types of cells indicating that a functional PPARγ protein is present in differentiated bMSC [41,42]. The absence of PPARγ signal in the control bMSC at the same stage may be the consequence of the reduced levels of *PPAR*γ mRNA detected in these cells, which were similar to the undifferentiated state at Day 0 of culture (0.73-fold expression of the undifferentiated bMSC). In contrast to *PPAR*γ, levels of *AP2* mRNA were up-regulated at Days 12 and 18 of culture in differentiated bMSC compared to controls. Adipogenesis was confirmed at Day 18 of culture by detection of lipid vacuoles using oil red staining. Overall these results indicate that bMSC from fetal BM conserve adipogenic potential under in vitro culture conditions; however, these cells may also present variations in the expression of some adipogenic markers described in postnatal MSC.

The levels of *NANOG* and *OCT4* mRNA were analyzed with the aim to evaluate the state of multipotency in bMSC during mesenchymal differentiation. NANOG and OCT4 regulate the maintenance of pluripotent state in embryos and derived cells in most mammalian species [43,44]. Both transcription factors had been proposed to play a similar role in adult stem cells [45]. However, recent reports have questioned this role under in vitro conditions [46]. Our analyses by Q-PCR using two different sets of primers were unable to detect *OCT4* mRNA both in undifferentiated and differentiated bMSC (data not shown). The lack of OCT4 expression has also been reported in MSC derived from human BM [13]. In our study, the pattern of *NANOG* mRNA changed according to lineage differentiation. While *NANOG* mRNA levels were not affected under adipogenic culture; the osteogenic and chondrogenic conditions induced higher and lower expression of this gene, respectively. These results agree with data in human MSC where expression of NANOG was associated to adaptation to in vitro cell growth conditions [13]. Moreover, these results suggest that *NANOG* expression is significantly affected by culture conditions of bMSC.

## Conclusions

Our data generated by quantitative and qualitative analyses support the capacity for multilineage differentiation of bone marrow MSC isolated from abattoir-derived bovine fetuses. The expression patterns of linage-specific markers in differentiating bMSC were similar to previously reported for bMSC derived from other sources. However, expression patterns of pluripotent markers mRNA *OCT4* and *NANOG* were different from previously reported in other species, suggesting that variation may occur according to animal species or culture conditions. The osteogenic differentiation of bMSC was affected by ascorbic acid and FBS concentrations in culture media. The simplicity of isolation and the potential to differentiate into several cell types lays the foundation for bone marrow MSC isolated from abattoir-derived bovine fetuses, as an alternative source of MSC for investigation of biology and eventual applications for regenerative therapy in human and veterinary medicine.

## Methods

All procedures have been approved by the Bioethical Committee of the National Commission for Scientific and Technology Research (Fondecyt).

### Isolation and culture of bMSC from fetal bone marrow

Bone marrow was aspirated from bovine fetuses (n = 10; 7–8 months of gestation) collected at a local abattoir. The marrow was drawn from femoral marrow cavity into syringes containing high glucose Dulbecco’s Modified Eagle Medium (DMEM, Gibco, Grand Islands, NY, USA) supplemented with 10% fetal bovine serum (FBS), 1000 U heparin, 100 U/ ml penicillin and 100 μg/ml streptomycin. Bone marrow samples were washed twice with phosphate-buffered saline (PBS) and twice with DMEM. Then cells were plated in DMEM (high glucose) supplemented with 10% FBS, 100 U/ml penicillin, 100 μg/ml streptomycin and 0.25 μg/ml amphotericin B. Cells were incubated at 38°C in a humidified atmosphere containing 5% CO_2_. Non-adherent cells were removed by changing the culture medium after 2 days. Following the initial 2 days, the medium was changed every 2–3 days. After 4 to 5 passages, cells were gently harvested when 90% confluent using 0.25% trypsin in 0.1% EDTA. Thereafter, cell number was determined at all consecutive passages and time of population doubling was calculated until culture passage 7. Following determination of cell viability, cells were used to initiate differentiation experiments.

### Osteogenic differentiation

Cells (5×10^4^/cm^2^) isolated from 3 fetuses were plated in T-25 culture dishes either in control (three replicates) or differentiation (three replicates) medium and cultured as described above for a 27-Day experiment. Control medium consisted of DMEM (high glucose) supplemented with 10% FBS, 100 U/ml penicillin, 100 μg/ml streptomycin and 0.25 μg/ml amphotericin B. Differentiation medium consisted of control medium supplemented with 100 nM dexamethasone, 10 mM sodium β-glycerophosphate, 0.05 mM ascorbic acid (all from Sigma-Aldrich, St. Louis, MO, USA). Cells were cultured for 24 days, with the medium being changed every 2 days. On Day 24, 10 nM of 1, 25-dihydroxyvitamin D_3_ (Vitamin D, Sigma) was added to differentiation medium until Day 27 of culture. Samples were obtained at Days 0, 8, 16, 24 and 27 and analyzed for *GAPDH*, β*-ACTIN, OC, OCT4* and *NANOG* expression by Q-PCR. The level of osteogenic differentiation was also analyzed at Day 27 of differentiation by immunodetection of OC protein, quantification of ALP activity and visualization of von Kossa staining of mineralized materials in the cell culture. In order to analyze the effect of various concentrations of ascorbic acid (0, 0.1, 0.01, and 0.001 mM) and FBS (0, 1, 5, and 10%) on bMSC osteogenic differentiation, cells were cultured under the same conditions described previously. The osteogenic differentiation in MSC was evaluated on Days 0 and 27 using the analyses described above.

### Chondrogenic differentiation

Cells (1×10^6^) isolated from 3 fetuses were resuspended into 1 ml of control (three replicates) or differentiation (three replicates) medium, transferred into 15-ml tubes and centrifuged at 500 g for 5 min. Control medium consisted of DMEM (high glucose) supplemented with 100 U/ml penicillin, 100 μg/ml streptomycin and 0.25 μg/ml amphotericin B. Differentiation medium consisted in control medium supplemented with 10% ITS (6.25 μg/ml insulin, 6.25 μg/ml transferrin, 6.25 μg/ mL selenious acid), 1 mM pyruvate, 50 μg/ml ascorbate 2-phosphate, 0.1 μM dexamethasone, and 8 ng/ml TGFβ1 (R&D Systems, MN, USA). Pellets were cultured for 21 days under 38°C in a humidified atmosphere containing 5% CO_2._ Samples were obtained at seven-day intervals for a total of 21 days and analyzed for *GAPDH*, β*-ACTIN, ACAN*, *COL2A1*, *SOX9, OCT4* and *NANOG* expression by Q-PCR. The level of chondrogenic differentiation was also analyzed at Day 21 of differentiation by immunodetection of ACAN protein and visualization of glycosaminoglycan formation using alcian blue staining in micromass histological sections.

### Adipogenic differentiation

Cells (2–5 7× 10^3^/cm^2^) isolated from 3 fetuses were seeded in control (three replicates) or differentiation (three replicates) medium. Control medium consisted of DMEM (high glucose) supplemented with 10% FBS, 100 U/ml penicillin, 100 μg/ml streptomycin and 0.25 μg/ml amphotericin B. Differentiation medium consisted in control medium supplemented with 10% FBS, 1 μM dexamethasone, 0.5 mM indomethacin, 10 μg/ml insulin and 100 mM 3-isobutyl-1-methylxanthine (all from Sigma). Cells were cultured in differentiation medium for 3 days and then in differentiation maintenance medium containing DMEM (high glucose), 10% FBS and 10 μg/ml insulin for 3 additional days in a total experimental period of 18 days. Samples were obtained at six-day intervals for 18 days and analyzed for *GAPDH*, β*-ACTIN*, *PPAR*γ, *AP2*, *OCT4* and *NANOG* expression by Q-PCR. The level of adipogenic differentiation was also analyzed at Day 18 of differentiation by immunodetection of PPARγ protein and visualization of fat aggregates in cultured cells using Oil Red staining.

### RNA Extraction and cDNA synthesis

Approximately 3 × 10^5^ MSC were collected and immediately fixed in RLT buffer (Qiagen, Incorporated, Valencia, CA). Total RNA was extracted using RNeasy Mini kit (Qiagen) according to the manufacturing’s instructions. The concentration and purity of the RNA in each sample were determined using spectrophotometry (BioRad Laboratories, Hercules, CA, USA). Total RNA was eluted in 30–50 μl of RNase free water. Samples were subjected to RT-PCR using a Brilliant II SYBR Green RT-PCR kit (Agilent Technologies, Santa Clara, CA, USA). The reaction protocol consisted of incubation for 5 min at 25°C, 15 min at 42°C, 5 min at 95°C and hold at 4°C using a DNA engine PCR thermocycler (Bio-Rad).

### Quantitative-PCR

Real-time PCR primers were designed using PrimerExpress software (Applied Biosystems Incorporated, Foster City, CA) (Table 1). Equivalence of amplification efficiencies among all primer-probe sets was confirmed using serial 3-fold dilutions of differentiated MSC cDNA. Each RT-PCR reaction (25 μl) contained the following: 2X Brilliant II SYBR Green QPCR master mix (12.5 μl), diluted reference dye (0.375 μl), target forward primer (200 nM), target reverse primer (200 nM), cDNA synthesis reaction (2 μl) and nuclease-free PCR-grade water to adjust final volume. The PCR amplification was carried out in StepOne Real Time PCR System (Applied Biosystems). Thermal cycling conditions were 95°C for 10 min, followed by 40 repetitive cycles at 95°C for 30 sec and 60°C for 1 min. As a normalization control for RNA loading, parallel reactions in the same multiwell plate were performed using glyceraldehyde-3-phosphate dehydrogenase (*GAPDH*) or β*-ACTIN* as a target. Quantification of gene amplification was made following Q-PCR by determining the threshold cycle (C_T_) number for SYBR fluorescence within the geometric region of the semilog plot generated during PCR. Within this region of the amplification curve, each difference of one cycle is equivalent to a doubling of the amplified product of the PCR. The relative quantification of the target gene expression across treatment was evaluated using the comparative ∆∆C_T_ method. The C_T_ value was determined by subtracting the most stable endogenous gene C_T_ value (*GAPDH*, osteogenesis and chondrogenesis; β*-ACTIN*, adipogenesis) from the target C_T_ value of the sample. Calculation of ∆∆ C_T_ involved using target gene expression on Day 0 (sample with the highest CT value or lowest target expression) as an arbitrary constant to subtract from all other C_T_ sample values. Relative target mRNA expression for differentiation markers was calculated as fold changes in relation to Day 0 sample and expressed as 2^-∆∆CT^ value. Relative mRNA expression for cell surface markers was calculated as fold changes in relation to CD34 mRNA levels and expressed as 2^-∆∆CT^ value.

**Table 1:** Sequence of primers used for Q-PCR analysis

** Gene**	**Sense**	**Antisense**	**Accession number**
*GAPDH*	5′ CCTTCATTGACCTTCACTACATGGTCTA	5′ TGGAAGATGGTGATGGCCTTTCCATTG	NM 001034034.2
β*ACTIN*	5′CGCACCACTGGCATTGTCAT	5′TCCAAGGCGACGTAGCAGAG	K00622.1
*CD34*	5′TGGGCATCGAGGACATCTCT	5′GATCAAGATGGCCAGCAGGAT	AB021662
*CD45*	5′CCTGGACACCACCTCAAAGCT	5′TCCGTCCTGGGTTTTATCCTG	NM 001206523
*CD73*	5′TGGTCCAGGCCTATGCTTTTG	5′GGGATGCTGCTGTTGAGAAGAA	BC114093
*CD90*	5′CAGAATACAGCTCCCGAACCAA	CACGTGTAGATCCCCTCATCCTT	BC104530
*CD105*	5′CGGACAGTGACCGTGAAGTTG	5′TGTTGTGGTTGGCCTCGATTA	NM 001076397
*OC*	5′ TGACAGACACACCATGAGAACCC	5′ AGCTCTAGACTGGGCCGTAGAAG	EF673278.1
*CD73*	5′AATGGCACGATTACCTGGGA	5′GGGAGGATCACCTTGTACAC	BT026240.1
*ACAN*	5′ CACTGTTACCGCCACTTCCC	5′ GACATCGTTCCACTCGCCCT	NM 173981.2
*COL2A1*	5′ ATCCATTGCAAACCCAAAGG	5′ GACATCGTTCCACTCGCCCT	NM 001113224.1
*SOX9*	5′CATGAAGATGACCGACGAG	5′ CGTCTTCTCCGTGTCGGA	AF278703.1
*AP2*	5′ CTGGCATGGCCAAACCCA	5′ GTACTTGTACCAGAGCACC	NM 174314.2
*PPAR*γ	5′ CGCACTGGAATTAGATGACAGC	5′ CACAATCTGTCTGAGGTCTGTC	BC116098.1
*NANOG*	5′ GTGTTTGGTGAACTCTCCTG	5′ GGGAATTGAAATACTTGACAG	NM 001025344.1
*OCT4* (1)	5′ ACACTCGGACCACGTCTTTC	5′ CGCATGGGTACCAGTGCACCTT	AF022987
*OCT4* (2)	5′ GTTCTCTTTGGAAAGGTGTTC	5′ ACACTCGGACCACGTCTTTC	AY490804.1

(1) and (2) represents sets of primers used for *OCT4* mRNA amplification.

### Immunofluorescence

Differentiated MSC were cultured in 35-mm dishes, fixed in a 4% paraformaldehyde (PAF) for 10 min and stored at 4°C under PBS. Cells were then washed twice in PBS twice and blocked in donkey serum (Sigma-Aldrich) for 30 min at RT. Cells were incubated over-night at 4°C with primary mouse monoclonal (OC) or goat polyclonal (ACAN or PPARγ) antibodies (1:50; Santa Cruz Biotechnology, Santa Cruz, CA, USA) diluted in donkey serum. After three washes with PBS, cells were incubated with goat anti-mouse or anti-goat IgG conjugated to FITC (1:200 in donkey serum). Then cells were again washed three times in PBS and mounted under coverslips in a solution containing 4′, 6-diamidino-2-phenylindole (Santa Cruz Biotechnology). Samples were examined under epifluorescence and the results captured by digital photomicroscopy (Olympus, Tokyo, Japan).

Cartilage pellet preparations obtained from chondrogenic differentiation were fixed in 4% PAF for 20 min and stored in PBS at 4°C until processing. Pellet preparations were pre-embedded in 4% agar (Bacto-agar, Difco Lab, Detroit, MI) under a stereomicroscope. Specimens were trimmed, embedded in paraffin and sectioned at 5–7 μm using a microtome. Tissue sections were mounted on adhesive slides (Newcomer Supply, Middleton, WI) and incubated overnight at 37°C. Mounted tissues were deparaffinized in xylene and rehydrated in serial alcohol solutions. Tissue sections were then rinsed two times in 0.1 M PBS (pH 7.4) and blocked in 2.5% donkey serum for 15 min. ACAN was specifically detected by overnight incubation at room temperature with primary antibody (1:50) diluted in 1.5% donkey serum solution (Santa Cruz Biotechnology). After two washes in 0.1 M PBS (pH 7.4), bound primary antibody was detected goat anti-mouse IgG conjugated to FITC (1:200 in donkey serum). Then cells were again washed three times in PBS and mounted under coverslips in a solution containing 4′, 6-diamidino-2-phenylindole (Santa Cruz Biotechnology) for nuclei visualization. Samples were examined under epifluorescence and the results captured by digital photomicroscopy (Olympus, Tokyo, Japan). Neighboring sections were used for the following experimental controls: 1) positive control using cartilaginous tissue, 2) replacement of the primary antibody with non-immune serum, 3) omission of primary and secondary antibody.

### Alkaline phosphatase

Activity of ALPL was detected in bMSC cultured under different osteogenic conditions after day 27 of culture using a Calbiochem kit (EMD Biosciences, CA, USA). Each analysis included a positive control (ALP) and a negative control (triton). bMSC were permeabilized using 0.2% triton for 20 min at 38°. Each reaction contained 50 μl of cell lysate, 100 μl of ALP substrate (p-NPP), 40 μl p-NPP concentrate, 1560 μl of dH2O and 400 μl of p-NPP buffer. Reaction tubes were analyzed at 1, 20, 25 and 30 min under 415 nm of absorbance using a spectrophotometer.

### von Kossa staining

Mineral deposits in differentiated and control CMM at day 27 of culture were detected using von Kossa staining. bMSC cultured in T-25 dishes were incubated with 5% solution of AgNO_3_ under darkness. Then cells were washed with distilled water (dH2O) and exposed to light. Cells were counterstained after incubation with 2% safranin solution for 3 min. Samples were examined under bright field microscope and the results captured by digital photomicroscopy (Olympus, Tokyo, Japan).

### Alcian blue staining

Cartilage pellets were fixed with 10% PAF for 2 h and then stored in PBS at 4°C until processed. Then pellets were paraffin embedded, sectioned at 5 μm sections and mounted on glass slides. Sagittal sections were deparaffinized, rehydrated in serial alcohol solutions and incubated in 0.5% alcian blue staining for 10 min. Samples were examined under bright field microscope and the results captured by digital photomicroscopy (Olympus, Tokyo, Japan).

### Oil red staining

Lipid vacuoles were detected in differentiated and controls bMSC after 18 days of adipogenic differentiation using oil Red staining. bMSC were fixed with 4% PAF for 1 h. Then cells were incubated in 0.2% oil red for 2 h, washed with dH2O and counterstained with hematoxylin. Samples were examined under bright field microscope and the results captured by digital photomicroscopy (Olympus, Tokyo, Japan).

### Data analysis

Values of gene expression from three different replicates were transferred to a spreadsheet and then analyzed using Infostat (version 2008). Data was normalized to logarithmic scale in base 10 for normality and mean values for each replicate were compared by one-way ANOVA. Gene expression values for each day of culture and cell surface markers, and PD values were compared using Dunnet test. Significant differences (P < 0.05) between days of culture and between treatments and controls were analyzed using Duncan’s multiple comparison test.

## Abbreviations

bMSC: Bovine mesenchymal stem cell; CD73: ecto-5′-nucleotidase; CD90: Thy-1; CD105: endoglin; CD45: Protein tyrosine phosphatase; C: receptor type; CD34: CD34 molecule; OC: Osteocalcin; AA: Ascorbic acid; CD73: Ecto-5′-nucleotidase; FBS: Fetal bovine serum; ACAN: Aggrecan; COL2A1: Collagen-2A1; SOX9: SRY (sex-determining region Y) box 9; AP2: fatty acid-binding protein 2; PPARγ: Peroxisome proliferation-activated receptor; Q-PCR: Quantitative-polymerase chain reaction; VK: von Kossa; OR: Oil red.

## Competing interests

The authors declare that they have no competing interests.

## Authors’ contributions

YC, MO and DA performed cell isolation and culture expansion, differentiation assays, statistical analyses and helped in the manuscript drafting. FD participated in the isolation and expansion of the cells and in gene expression analyses. MSF helped in the cell culture staining and in the manuscript drafting. OAP conceived and designed the study, analyzed the data and drafted the manuscript. All authors read and approved the final manuscript.
